# Disease severity is associated with the use of complementary medicine to treat or manage type-2 diabetes: data from the 2002 and 2007 National Health Interview Survey

**DOI:** 10.1186/1472-6882-12-193

**Published:** 2012-10-22

**Authors:** Richard L Nahin, Danita Byrd-Clark, Barbara J Stussman, Nilesh Kalyanaraman

**Affiliations:** 1 National Center for Complementary and Alternative Medicine, National Institutes of Health, 6707 Democracy Blvd, Bethesda, MD, 20892-5475, USA; 2 Social & Scientific Systems, Inc, 8757 Georgia Avenue, Silver Spring, MD, 20910, USA

**Keywords:** Complementary medicine, Diabetes, Disease severity, Logistic regression, Survey

## Abstract

**Background:**

The overall prevalence of complementary medicine (CM) use among adults in the United States with diabetes has been examined both in representative national samples and in more restricted populations. However, none of these earlier studies attempted to identify predictors of CM use to treat diabetes among the populations sampled, nor looked for a relationship between CM use and diabetes severity.

**Methods:**

Combining data from the 2002 and 2007 National Health Interview Survey (NHIS), we constructed a nationally representative sample of 3,978 U.S. adults aged ≥18 years with self-reported diabetes. Both the 2002 and 2007 NHIS contained extensive questions on the use of CM. We used logistic regression to examine the association between diabetes severity and overall CM use, as well as the use of specific categories of CM.

**Results:**

In adults with type-2 diabetes, 30.9% used CM for any reason, but only 3.4% used CM to treat or manage their type-2 diabetes versus 7.1% of those with type-1 diabetes. Among those using CM to treat/manage their type-2 diabetes, 77% used both CM and conventional prescription medicine for their diabetes. The most prevalent types of CM therapies used were diet-based interventions (35.19%, S.E. 5.11) and non-vitamin/non-mineral dietary supplements (33.74%, S.E. 5.07). After controlling for sociodemographic factors, we found that, based on a count of measures of diabetes severity, persons with the most severe diabetes had nearly twice the odds of using CM as those with less severe disease (OR=1.9, 95%CI 1.2-3.01). Persons who had diabetes 10 years or more (OR=1.66, 95%CI 1.04-3.66) and those that had a functional limitation resulting from their diabetes (OR=1.74, 95%CI 1.09-2.8) had greater odds of using CM than those not reporting these measures. No significant associations were observed between overall CM use and other individual measures of diabetes severity: use of diabetic medications, weak or failing kidneys, coronary heart disease, or severe vision problems.

**Conclusions:**

Our results demonstrate that individuals with more severe diabetes are more likely to use CM independent of sociodemographic factors. Further studies are essential to determine if CM therapies actually improve clinical outcomes when used to treat/manage diabetes.

## Background

Self-management is the cornerstone of overall diabetes management [1]. Self-management of type-2 diabetes requires complex, continual and demanding self-care behaviors, including dietary control, exercise and frequent medication. More active self-management is generally believed to result in better metabolic control and higher quality of life [2] while also being more cost effective than standard pharmaceutical therapies alone [3,4]. Yet failure to follow treatment recommendations is reported as a serious and widespread problem in patients with type-2 diabetes and believed to lead to diabetic complications such as blindness, poor wound healing, neuropathy and kidney failure.

Hernanderz et al. [5] and Paterson and Thorne [6] have demonstrated that learning to master self-care in diabetes is a process where the person attempts a variety of self-care strategies according to her or his unique circumstances until discovering what is effective for their own lifestyle and contextual situation. It has been proposed that individuals with diabetes view complementary medicine (CM) as another option for their self management along with conventional options [7], and that individuals highly motivated to control their diabetes will try multiple therapies available to them including CM [8]. This is consistent with numerous observations that CM therapies are predominately used as complements to conventional care rather than in lieu of conventional care [9,10].

The overall prevalence of CM use among adults in the United States with diabetes has been examined both in representative national samples [11-13] and in more restricted populations [7,8]. Prevalence rates in these studies varied from about 30% to 70% depending on the definition of CM used. Much lower rates are observed when examining the prevalence of CM use specifically to treat or manage diabetes – from about 20% of all adults with diabetes in 1997 [11] to about 6.7% of all adults with diabetes in 2002 (recalculated from [12]). None of these studies attempted to identify predictors of CM use to treat diabetes among the populations sampled, nor looked for a relationship between CM use and diabetes severity.

In the general population, a number of characteristics are associated with CM use: gender (being female), race/ethnicity (being non-Hispanic White), age (being middle-aged), and education (having a college degree) to name a few [10,14,15]. Other predictors of CM use include poor perceived health status and the presence of multiple health complaints [14-17]. Thus, one might hypothesize that individuals with diabetes are more likely to use CM if they have more severe diabetes and/or one or more complications associated with the disease. However, there are also data from the general population showing that CM is associated with a number of positive health behaviors that would be part of a wellness lifestyle [18] including regular levels of exercise [15], nonuse of tobacco [15,19,20], nonuse or moderation in use of alcohol [15,21], healthy diet choices [21] and preventive screening [22]. It may also be that individuals oriented toward a wellness lifestyle will be more likely to use CM for their diabetes. The present study seeks to address these two, not mutually exclusive hypotheses through analysis of data from the National Health Interview Survey (NHIS). In both 2002 and 2007, the NHIS included extensive questions on the use of a wide range of CM therapies. The NHIS also contained a limited number of questions on the reasons why an individual might have chosen to use a given CM therapy. We analyzed the responses to two of these questions to examine reasons for use of CM among individuals who used CM to treat/manage their diabetes.

## Methods

### Data source

The data used in this study come from the 2002 and 2007 NHIS. The NHIS is an annual survey of the health of the U.S. civilian, non-institutionalized population conducted by the National Center for Health Statistics, Centers for Disease Control and Prevention. The survey uses a multi-stage clustered sample design, and oversampled non-Hispanic black and Hispanic persons in both 2002 and 2007, and Asians in 2007 to allow for more accurate national estimates of health for these increasing minority populations.

The survey contains four main modules: Household, Family, Sample Child, and Sample Adult. The first two modules collect health and sociodemographic information on each member of all families residing within a sampled household, while the Sample Adult file includes more specific information (e.g., health and health-related information) obtained from a randomly chosen adult aged 18 years or older in the household. The NHIS also contains supplemental questions/modules on a yearly basis. Both the 2002 and 2007 NHIS included a module that asked about the use of various CM therapies including practitioner-based therapies (e.g., chiropractic and osteopathic manipulation, massage therapy, acupuncture, etc.) and self-care therapies (e.g., dietary supplements, yoga, meditation, etc.). The consistency in the sample design, weighting, and CM modalities covered in the 2002 and 2007 NHIS made it possible to combine data across the two samples.

In 2002, NHIS interviews were completed in 36,161 households, which yielded 93,386 persons in 36,831 families and 31,044 Sample Adults. The final household response rate was 89.6% and final sample adult response rate was 74.3%. In 2007, NHIS interviews were completed in 29,266 households, which yielded 75,764 persons in 29,915 families and 23,393 Sample Adults. The final household response rate was 87.1% and the final 2007 sample adult response rate was 67.8%.

Participants were asked “other than during pregnancy, have you ever been told by a doctor or health professional that you have diabetes or sugar diabetes?” Based on this question, in 2002, 2186 sample adults (7.04% unweighted; 6.51% weighted) were classified as having diabetes. Of those, 2159 (98.8% unweighted; 98.6% weighted) had valid responses to the series of CM-related questions. In 2007, 2036 of sample adults (8.7% unweighted; 7.74 weighted) reported having diabetes; of those, 1991 (97.8% unweighted; 97.9% weighted) had valid response to the series of CM questions. Because participants in NHIS were not categorized as having type-1 or type-2 diabetes, participants with diabetes diagnosed at age <25 and who were currently treated with insulin were excluded from subsequent analysis as probably having type-1 diabetes (N=172; Table 1). These criteria are based on those used in previous analyses of type-2 diabetes using NHIS data [23,24]. Thus our final sample for analysis included 3978 participants from 2002 and 2007 combined who were coded as having type-2 diabetes.

**Table 1:** Characteristics of sample adults with Type 1 and Type 2 diabetes: data from the 2002 and 2007 NHIS

	Type 1 N=172*			Type 2 N=3978*		
**% of adult sample (S.E.)†**	0.32 (0.03)			6.83 (0.14)		
**% of individuals with diabetes (S.E.)†**	4.5 (.4)			95.5 (.4)		
**Weighted sample**	689,758			14,642,272		
	N*	%	S.E^.‡^	N*	%†	S.E.^‡^
**Count of diabetes severity measures**^**§**^						
**0-2**	**87**	49.99	4.42	2835	76.66	0.84
**>=3**	**84**	50.01	4.79	945	23.34	0.84
**DM Duration**						
1-< 5 years	10	5.04	2.26	1393	37.24	0.94
5-<10 years	12	6.72	2.13	856	23	0.95
>= 10 years	150	88.25	3.03	1610	39.77	1
**Diabetes Medication use**						
None	0	0		580	16.08	0.76
Any use	172	100		3387	83.92	0.76
**Functional limitation**						
No	125	76.47	3.87	3209	83.13	0.68
Yes	46	23.53	3.87	721	16.87	0.68
**Weak or failing Kidneys**						
No	149	85.55	3.53	3690	93.25	0.5
Yes	23	14.45	3.53	279	6.75	0.5
**Severe Vision Problem**						
No	118	69.47	4.43	3026	78.86	0.79
Yes	53	30.53	4.43	910	21.14	0.79
**Gender**						
Male	73	44.15	4.49	1788	49.73	1.01
Female	99	55.85	4.49	2190	50.27	1.01
**Age group**						
Age 18-44 yrs	95	57.82	4.73	473	12.68	0.7
Age 45-64 yrs	56	32.05	4.42	1726	47	0.98
Age 65+ yrs	21	10.13	2.43	1779	40.32	0.9
**Education**						
Less than high school	36	21.39	4.16	1228	27.24	0.86
At least high school	135	78.61	4.16	2695	72.76	0.89
**Race/ethnicity**						
Non-Hispanic white	101	64.7	4.39	2255	65.26	0.99
Non-Hispanic black	32	15.67	3.38	822	15.69	0.68
Hispanic	30	14.85	3.07	687	12.48	0.62
Other races	9	4.78	1.75	214	6.56	0.61
**Region of residence**						
Northeast	24	15.36	3.68	661	16.06	0.78
Midwest	44	25.14	3.9	868	23.35	1.02
South	69	40.82	4.71	1617	40.52	1.09
West	35	18.69	3.23	832	20.07	0.9
**Health Insurance**						
Public Insurance	56	31.61	4.51	1429	30.17	0.9
Private Insurance	99	59.06	4.55	2172	60.21	1
No Insurance	16	9.33	2.81	366	9.62	0.62
**Other health conditions**^||^						
0-2	139	83.49	139	2774	70.1	0.91
3 or more	33	16.51	33	1204	29.9	0.91
**BMI Level**						
BMI 0 - < 25	63	37.24	4.64	687	16.01	0.67
BMI 25 - < 30	36	19.65	3.65	1251	31.96	0.94
BMI >= 30	73	43.11	4.73	2040	52.03	0.96
**Hypertension**						
No	97	57.32	4.71	1614	42.6	0.98
Yes	75	42.68	4.71	2350	57.4	0.98
**Coronary heart disease**						
No	154	89.57	2.69	3489	88.12	0.64
Yes	19	10.43	2.69	458	11.88	0.64
**Smoking status**						
Current	30	20.42	4.25	618	16.23	0.76
Former smoker	34	18.09	3.2	1382	35.49	0.98
Never smoker	107	61.49	4.73	1953	48.28	0.99
**Alcohol use**						
Never/none	56	30.21	4.27	1242	29.01	0.91
Any	115	69.97	4.27	2703	70.99	0.91
**Anxiety or Depression**						
No	124	72.26	4.39	2900	73.58	0.88
Yes	48	27.74	4.39	1068	26.42	0.88
**Vigorous leisure activity**						
Unable to do or none	110	66.54	4.56	2877	77.44	0.95
Any per week	50	33.46	4.56	742	22.55	0.95
**Perceived health status**						
Fair/poor	84	53.14	4.61	1771	43.28	1
Exc/VG/Good^¶^	88	46.86	4.61	2204	56.72	1
**Any CM Use to Treat or Manage Diabetes**	13	7.06	2.27	141	3.43	0.37

* The unweighted number of participants belonging to each category.

† The denominator used in the calculation of percentages was the number of adults with type-2 Diabetes, defined as all participants with diabetes excluding those who were both diagnosed at age <25 and were currently being treated with insulin, who were classified as having type-1 diabetes. Estimates were age adjusted using the projected 2000 U.S. population as the standard population and using four age groups: 18–24 years, 25–44 years, 45–64 years, and 65 years and over.

‡ Standard Errors.

§ Based on the following six measures: 5 or more years since time of diagnosis, use of insulin or oral hypoglycemic drugs, at least one functional limitation attributed to the diabetes, weak or failing kidneys, Coronary Heart Disease, and severe vision problems.

|| Includes all health conditions included in the NHIS except weak or failing kidney, severe vision problems, coronary heart disease, hypertension, and anxiety or depression.

¶ Excellent/Very Good/Good.

The surveys were approved by the National Center for Health Statistics Institutional Review Board. Verbal or written consent was obtained from all survey participants.

#### Dependent variable

The NHIS applied the definition of CM as used by the National Institutes of Health, National Center for Complementary and Alternative Medicine at the time each survey was designed and fielded [16,17]. Administered to sample adults, the supplement asked a number of questions about the use of CM therapies within the past 12 months. CM use, the dependent variable for this study, was defined as use of any of the following in the past 12 months: acupuncture, Ayurveda, biofeedback, chelation therapy, chiropractic care, chiropractic or osteopathic manipulation, energy healing therapy/Reiki, folk medicine, hypnosis, massage therapy, movement based therapies (Feldenkrais, Alexander technique, Pilates, and Trager Psychophysical Integration), naturopathy, herbal supplements and other non-vitamin/non-mineral dietary supplements (NVNMDS), homeopathic treatment, diet-based therapies (Vegetarian diet, Macrobiotic diet, Atkins diet, Pritikin diet, Ornish diet and Zone diet, and South Beach diet), traditional healers, yoga, tai chi, qi gong, and relaxation techniques (meditation, guided imagery, progressive relaxation, and deep breathing exercises). For exploratory purposes, CM therapies were also grouped into four discrete categories: use of only one or more specific NVNMDS, use of only one or more types of diet-based therapies, use of all other CM therapies, and use of multiple CM therapies (for coding purposes, use of multiple NVNDMS or multiple diet-based therapies are counted once– e.g., use of two or more specific herbs would be coded once as use of NVNMDS; use of two or more specific diets would be coded once as use of CM diets.). This last category was included as a surrogate measure for heavy CM users.

#### Independent variables

The main independent variable was based on counts of six measures of diabetes severity: three direct measures of diabetes severity and three known diabetes complications. An individual reporting the presence of three or more of these individual measures was considered to have more severe diabetes versus someone reporting less than three. The three direct measures of greater diabetes severity were: 1) 5 or more years since time of diagnosis; 2) use of insulin or oral hypoglycemic drugs (diabetes prescription medications); and 3) at least one functional limitation attributed to the diabetes. The three diabetes complications counted were: 1) weak or failing kidneys; 2) severe vision problems; and 3) coronary heart disease. The items chosen for the count were based on the literature where measures of diabetes severity routinely included use of diabetes prescription drugs [25], time since diagnosis [25], and major complications of diabetes (even if self-reported) including kidney disease [25-27], retinal diseases [25-27] and coronary heart disease [25-27], but not co-morbid diseases that are not considered complications of diabetes.

The coding for weak/failing kidneys and coronary heart disease were based on “yes” responses to the following questions in the NHIS: “Have you ever been told by a doctor or other health professional that you have: coronary heart disease . . . weak or failing kidneys.” Individuals were coded as having severe vision problems if either they responded yes to the NHIS question “Do you have trouble seeing even when wearing glasses or contacts” OR had a functional limitation (see below) resulting from “vision problem/problem seeing”.

Also included in some of the published studies examining diabetes severity were diabetes-related functional limitations [27,28]. In the present study, the measure of functional limitation for diabetes was based on the following sequential questions from the NHIS core: “The next questions ask about difficulties you may have doing certain activities because of a HEALTH PROBLEM. By “health problem” we mean any physical, mental, or emotional problem or illness (not including pregnancy).” The questionnaire goes on to ask about 12 activities such as walking one-quarter mile, grasping small objects, pushing something heavy, or participating in various social activities. Difficulties with any of these 12 activities would elicit the following follow-up question: “What condition or health problem causes you to have difficulty with [problem 1, problem 2 or problem 3]”. If the participant reported that any specific functional limitation resulted from diabetes, or vision problems/problems seeing, for the current analysis they were coded as having a functional limitation associated with diabetes, or a severe vision problem, respectively.

When developing our list of diabetes severity measures to count, we explored the correlational relationships among our dichotomous variables through the tau statistic, which measures the amount of concordance and discordance among variables. For all 15 comparisons we made (each individual variable in the count vs. every other individual variable in the count), the calculated taus’s were all less than 0.2, indicating that there was substantial discordance among the variables (that is, relatively low collinearity).

The literature shows that while anxiety/depression is seen as a risk factor for diabetes in many individuals, it is a complication of diabetes in others [29,30]. As such, in sensitivity analysis, we also examined the impact of adding depression to the co-morbidities to be included in the diabetes severity count. The calculated taus’s comparing anxiety/depression to every other measures used in the count were all less than 0.2.

#### Control variables

Variables often associated with CM use among NHIS participants [15-17,31] were included in these analyses as control variables. These included sex, age (18–44; 45–64; 65+), education (<high school, HS or greater), race/ethnicity (non-Hispanic white, non-Hispanic black, Hispanic, other), region (Northeast, Midwest, South, West), health insurance status (no insurance, public insurance, private insurance), hypertension (yes, no), other health conditions (excluding weak/failing kidneys and severe vision problems, hypertension, and anxiety/depression; 0–2 versus 3 or more), perceived health status (fair/poor, excellent/very good/good), ability to perform vigorous leisure activity (unable to do, any per week), smoking status (never, former, or current), alcohol consumption (never/none, any), and BMI (0-<25 kg/m^2^; 25-<30 kg/m^2^; >=30 kg/m^2^). For our coding of alcohol use, we have employed the CDC definition of alcohol abstainer based on responses to two questions: “In ANY ONE YEAR, have you had at least 12 drinks of any type of alcoholic beverage?” and “In your ENTIRE LIFE, have you had at least 12 drinks of any type of alcoholic beverage?”.

### Reasons for CM use

We conclude these analyses by presenting data on whether individuals who use CM to treat/manage their type-2 diabetes are dissatisfied with their conventional care. Specifically, we analyze data from two questions in the NHIS: “DURING THE PAST 12 MONTHS, did you use {given CM therapy}…Because medical treatments did not help; Because medical treatments were too expensive.” Chi square analysis was used to identify significant associations between these reasons and the various measures of diabetes severity.

### Statistical analysis

Descriptive statistics (means, percentages) for the characteristics of individuals coded as having type-2 diabetes were calculated for any CM use, and for each of the four CM categories. Chi-square analysis was used to test bivariate associations between the independent/control variables and any CM use, as well as for each of the four CM categories.

Multiple logistic regression was used to assess the relationships between the count measure of diabetes severity and use of CM in the past 12 months, after adjusting for control variables. Only control variables associated with CM use at p< 0.1 level in chi-square analysis were retained as control variables in the adjusted regression model. In addition, to control for any secular trends, the year of the survey (2002 or 2007) was forced into all adjusted models. As secondary analyses, we also individually explored the associations between each of the six measures of diabetes severity (plus anxiety/depression in sensitivity analysis) with use of CM.

For the multiple logistic regression models, there was no evidence of collinearity; inspections of tolerance values, condition indices, and variance inflation factors, suggested we had employed properly specified heteroskedastic models.

All estimates, including those of CM prevalence, were generated using SUDAAN software (version 10, Research Triangle Institute, Research Triangle Park, N.C.) that accounts for complex sample designs such as that used by the NHIS. To ensure representation of the U.S., civilian, non-institutionalized population age 18 years and over, all estimates (percentages, odds ratio, standard errors, 95% confidence limits) were weighted using the NHIS sample adult record weight.

### Sensitivity analyses

Based on previous analyses of NHIS data and other population-based data [23,24,32,33] and taking into account the growing rate of type-2 diabetes in youth, we had set our criteria for *probable* type-1 diabetes as participants with diabetes diagnosed at age <25 and who were currently treated with insulin. However, based on more recent data, it can be argued that a lower age cutoff is warranted [34-37]. Therefore, we have performed sensitivity analyses where we have reduced that age cutoff down in five-year increments, thereby theoretically capturing for analysis most, if not all, individuals with type-2 diabetes in our NHIS sample. Frequencies, chi sq and logistic regression data for this sensitivity analysis are presented.

## Results

We found that 7.2% (S.E. 0.14) of surveyed adults reported having physician diagnosed diabetes. This equates to roughly 15.3 million adults in the United States. Of these, 95.5% (S.E. 0.4), or 14.6 million, were coded as having type-2 diabetes. The majority of individuals with type-2 diabetes had the disease longer than 5 years, were taking either insulin or oral hypoglycemic medication, did not have a functional limitation related to diabetes, did not have weak/failing kidneys or severe vision problems, were at least 45 years old, had greater than a high school education, were Non-Hispanic whites, had private health insurance, were overweight or obese, had hypertension but not coronary heart disease, had smoked tobacco and consumed alcohol at some point in their lives, were not depressed or anxious, and were unable to perform vigorous leisure activity but nevertheless considered themselves in at least good health (Table 1). About equal numbers of men and women had type-2 diabetes. In contrast, individuals with type-1 diabetes predominately had their diabetes more than 10 years, were under 45 years of age, did not have a functional limitation related to diabetes, did not have weak/failing kidneys, severe vision problems, hypertension, coronary heart disease or depression, yet considered their health as only fair or poor (Table 1).

Consistent with the literature, we found that 30.9% (S.E. 0.96) of individuals with type-2 diabetes used CM for some reason, equating to roughly 4.4 million adults (data not shown). However, only 3.43% (S.E. 0.37) of individuals with type-2 diabetes used CM specifically to treat or manage their diabetes versus 7.06% of those with type-1 diabetes (Table 1). Among those using CM to treat/manage their type-2 diabetes, 63% have had their diabetes for more than five years, and 77% used both CM and conventional prescription medicine for their diabetes (Table 2). Only a quarter of individuals using CM to treat/manage their type-2 diabetes reported a functional limitation resulting from their diabetes, severe vision problems or anxiety/depression (Table 2). Very few individuals using CM for their diabetes reported weak/failing kidneys or coronary heart disease (Table 2).

**Table 2:** Characteristics of adults who used complementary medicine (CM)* to treat their type-2 diabetes

	**Did not use CM to treat/manage their diabetes**			**Used CM to treat/manage their diabetes**			
	**N**^**†**^	**Percent**^**‡**^	**S.E.**^**§**^	**N**^**†**^	**Percent**^**‡**^	**S.E.**^**§**^	
**All adults with type-2 diabetes**	3873	96.57	0.37	141	3.43	0.37	
	N^†^	Percent^||^	S.E. ^§^	N^†^	Percent^||^	S.E. ^§^	P-value^¶^
**Count of diabetes severity measures**^**#**^							
0-2 measures	2738	76.82	0.85	97	72.25	4.29	0.284
>=3 measures	904	23.18	0.85	41	27.75	4.29	
**Diabetes duration**							
1-< 5 years	1348	37.2	0.96	45	38.29	5.35	0.067
5-<10 years	835	23.28	0.98	21	15.11	3.58	
>= 10 years	1536	39.52	1.02	74	46.6	5.33	
**Diabetes medication use**							
None	554	15.83	0.77	26	23.08	4.9	0.16
Any	3273	84.17	0.77	114	76.92	4.9	
**Functional limitation**							
No	3105	83.34	0.7	104	77.34	3.7	0.104
Yes	684	16.66	0.7	37	22.66	3.7	
**Weak/failing kidneys**							
No	3561	93.28	0.49	129	92.38	2.76	0.741
Yes	268	6.72	0.49	11	7.62	2.76	
**Coronary heart disease**							
No	3359	87.95	0.66	130	92.97	2.27	0.044
Yes	447	12.05	0.66	11	7.03	2.27	
**Severe vision problems**							
No	2921	78.91	0.8	105	77.21	4.29	0.706
Yes	874	21.09	0.8	36	22.71	4.29	
**Anxiety or depression**							
No	2800	73.62	0.89	100	72.59	4.82	0.823
Yes	1027	26.38	0.89	41	27.41	4.82	
**Gender**							
Male	1725	49.83	1.04	63	46.95	4.79	0.56
Female	2112	50.17	1.04	78	53.05	4.79	
**Age group**							
Age 18-44 yrs	451	12.54	0.71	22	16.65	4.02	0.04
Age 45-64 yrs	1653	46.71	0.99	73	55.11	4.97	
Age 65+ yrs	1733	40.75	0.91	46	28.24	4.34	
**Education**							
Less than high school	1190	27.38	0.89	38	23.4	4.17	0.355
At least high school	2594	72.62	0.89	101	76.6	4.17	
**Income**							
$0-$34,999	2334	50.57	1.11	81	44.41	5.37	0.337
$35,000-$74,999	1035	32.05	1.15	40	29.08	5.03	
$75,000 +	468	17.38	0.99	20	26.51	5.8	
**Race/ethnicity**							
NH White	2183	65.46	0.99	72	59.5	5.46	0.059
NH Black	802	15.9	0.68	20	9.93	2.7	
Hispanic	650	12.25	0.64	37	19.09	3.42	
Other races	202	6.39	0.59	12	11.48	4.32	
**Region of residence**							
Northeast	648	16.27	0.78	13	10.09	3.28	0.01
Midwest	846	23.6	1.05	22	16.44	3.9	
South	1565	40.75	1.11	52	33.89	4.95	
West	778	19.38	0.9	54	39.58	5.47	
**Health insurance**							
Public insurance	1390	30.43	0.91	39	22.57	4.02	0.135
Private insurance	2096	60.09	1.02	76	63.55	4.91	
No insurance	342	9.47	0.63	24	13.88	3.46	
Other health conditions^**^							
0-2	2694	70.54	0.93	80	57.84	5.41	0.017
3 or more	1143	29.46	0.93	61	42.16	5.41	
**BMI level**							
BMI 0 - < 25	673	16.3	0.68	14	7.65	2.25	0.002
BMI 25 - < 30	1210	32.08	0.86	41	28.71	4.94	
BMI >= 30	1954	51.62	0.98	86	63.64	5.08	
**Hypertension**							
No	1544	54.41	1	70	48	5.19	0.297
Yes	2279	57.59	1	71	52	5.19	
**Smoking status**							
Current	605	16.4	0.77	13	11.61	3.99	0.441
Former smoker	1329	35.52	0.99	53	34.51	4.69	
Never smoker	1878	48.08	1	75	53.89	5.33	
**Alcohol use**							
Never/none	1202	29.19	0.94	40	23.97	3.91	0.21
Any	2604	70.81	0.94	99	76.03	3.91	
**Vigorous leisure activity**							
Unable to do or none	2784	77.82	0.95	93	67.08	5.79	0.086
Any per week	706	22.18	0.95	36	32.92	5.79	
**Perceived health status**							
Fair/poor	1715	43.74	1.02	56	30.35	4.58	0.012
Exc/VG/Good^††^	2119	56.26	1.02	85	69.65	4.58	

* CM use, the dependent variable for this study, was defined as use of any of the following in the past 12 months: acupuncture, Ayurveda, biofeedback, chelation therapy, chiropractic care, chiropractic or osteopathic manipulation, energy healing therapy/Reiki, folk medicine, hypnosis, massage therapy, movement based therapies, naturopathy, herbal supplements and other non-vitamin/non-mineral dietary supplements (NVNMDS), homeopathic treatment, diet-based therapies, traditional healers, yoga, tai chi, qi gong, and relaxation techniques.

† The unweighted number of participants belonging to each category.

‡ The denominator used in the calculation of percentages was the number of adults with type-2 Diabetes, defined as all participants with diabetes excluding those who were both diagnosed at age <25 and were currently being treated with insulin, who were classified as having type-1 diabetes. Estimates were age adjusted using the projected 2000 U.S. population as the standard population and using four age groups: 18–24 years, 25–44 years, 45–64 years, and 65 years and over”.

§ Standard Errors.

|| For each individual characteristic, the denominator used in the calculation of percentages was the number of adults with type-2 Diabetes for whom a response was recorded for the particular characteristic. Estimates were age adjusted using the projected 2000 U.S. population as the standard population and using four age groups: 18–24 years, 25–44 years, 45–64 years, and 65 years and over”.

¶ Chi-square analyses comparing individuals with Type-2 diabetes who used CM to treat/manage their diabetes versus those not using CM to treat/manage their diabetes.

# Based on the following six measures: 5 or more years since time of diagnosis, use of insulin or oral hypoglycemic drugs, at least one functional limitation attributed to the diabetes, weak or failing kidneys, Coronary Heart Disease, and severe vision problems.

** Includes all health conditions included in the NHIS except weak or failing kidney, severe vision problems, coronary heart disease, hypertension, and anxiety or depression.

†† Excellent/Very Good/Good.

Within the group using CM to treat or manage their diabetes, we observed that a number of sociodemographic and health status variables were associated with CM use at p<0.1 (Table 2): age, race/ethnicity, region of residence, number of co-morbid conditions, BMI level, the ability to perform vigorous leisure activity at least once per week and perceived health status. These variables were included in all adjusted logistic regression models as covariates.

### Diabetes severity

Table 3 shows the results from unadjusted and adjusted logistic regression models fitted for our count measure of disease severity. We found that more severe disease was associated with CM use, with someone exhibiting three or more measures of diabetes severity having almost twice the adjusted odds of using CM as someone with less than three measures. To explore this finding in more detail, we examined the association between CM use and each measure of diabetes severity contributing to the composite measure. Although no statistically significant associations were seen in the unadjusted analyses (Table 3), the odds ratio point estimates for use of diabetes prescription medications and coronary heart disease suggest negative associations with CM use, while the point estimates for the other four measures suggest positive associations with CM use. In the adjusted model, those individuals whose diabetes was diagnosed 10 or more years ago had 66% greater odds of using CM than those diagnosed within the last five years, while those individuals with a functional limitation resulting from their diabetes had 74% greater odds of using CM than individuals without a limitation (Table 3). Adjusting for covariates in the logistic regression model did not substantially change the odds ratio point estimates for the other measures of diabetes severity. Simultaneously adding all individual measures of diabetes severity to the adjusted model at the same time also did not substantially change any of these findings (data not shown).

**Table 3:** Associations of diabetes severity with use of complementary medicine (CM)* to treat/manage type-2 diabetes

	**UOR**^**†**^	**95% CI**^**‡**^	**AOR**^**§**^	**95% CI**^**‡**^
**Count of diabetes severity measures**^**||**^				
0-2 measures	ref		Ref	
>=3 measures	1.27	.83-1.94	1.90	1.2-3.01
**Individual measures of diabetes severity**				
**DM duration**				
1-< 5 years	ref		Ref	
5-<10 years	0.63	.34-1.18	0.70	.36-1.35
>= 10 years	1.15	.71-1.85	1.66	1.04-2.66
**Diabetes medication use**				
None	ref		Ref	
Any	0.63	.36-1.06	0.66	.36-1.19
**Functional limitation**				
No	ref		Ref	
Yes	1.47	.95-2.25	1.74	1.09-2.80
**Weak/failing kidneys**				
No	ref		Ref	
Yes	1.15	.53-2.47	1.46	.60-3.53
**Coronary Heart Disease**				
No	ref		Ref	
Yes	0.55	.28-1.10	0.87	.43-1.74
**Severe vision problems**				
No	ref		Ref	
Yes	1.1	.68-1.78	1.48	.85-2.57

* CM use, the dependent variable for this study, was defined as use of any of the following in the past 12 months: acupuncture, Ayurveda, biofeedback, chelation therapy, chiropractic care, chiropractic or osteopathic manipulation, energy healing therapy/Reiki, folk medicine, hypnosis, massage therapy, movement based therapies, naturopathy, herbal supplements and other non-vitamin/non-mineral dietary supplements (NVNMDS), homeopathic treatment, diet-based therapies, traditional healers, yoga, tai chi, qi gong, and relaxation techniques.

† unadjusted or crude odds ratio.

‡ 95% confidence interval.

§ Adjusted Odds Ratio. The model controls for socio-demographic and health status variables significantly associated (p < 0.1) with the dependent variables in Table 2: race/ethnicity, region of residence, number of co-morbid conditions, BMI level, the ability to perform vigorous leisure activity at least once per week and perceived health status, as well as for year of survey.

|| Based on the following six measures: 5 or more years since time of diagnosis, use of insulin or oral hypoglycemic drugs, at least one functional limitation attributed to the diabetes, weak or failing kidneys, Coronary Heart Disease, and severe vision problems.

### Control variables

Table 4 presents unadjusted and adjusted odds ratios for the control variables included in the regression models. In both the unadjusted and adjusted analyses, residing in the West versus other parts of the country, having an unhealthily high BMI (>=30), and having three or more comorbidities (other than weak/failing kidneys, severe vision problems, coronary heart disease, or hypertension) were significantly associated with higher CM use, while having a fair or poor perceived health status was associated with significantly lower CM use (Table 4). Whereas in the unadjusted analysis Hispanic respondents were found to have significantly higher odds of using CM than were non-Hispanic whites, those differences became non-significant in multivariate analyses. Similarly, after adjusting for the other variables, the negative relationships between CM use and being at least 65 years of age, and CM use and the inability to perform vigorous activity disappeared, though the point estimates remained above 1.0.

**Table 4:** Associations of socio-demographic and health status variables with use of complementary medicine (CM)* to treat/manage type-2 diabetes

	**UOR**^**†**^	**95% CI**^**‡**^	**AOR**^**§**^	**95% CI**^**‡**^
**Age group**				
Age 18–44 yrs	Ref		Ref	
Age 45–64 yrs	0.89	.48-1.63	1.16	.56-2.37
Age 65+ yrs	0.52	.27-.99	0.76	.36-1.59
**Race/ethnicity**				
NH White	Ref		Ref	
NH Black	0.69	.38-1.26	0.76	.39-1.46
Hispanic	1.71	1.05-2.81	1.39	.83-2.33
Other races	1.98	.83-4.72	1.49	.63-3.55
**Region of residence**				
Northeast	0.3	.14-.66	0.30	.14-.63
Midwest	0.34	.18-.65	0.32	.16-.62
South	0.41	.24-.68	0.40	.25-.65
West	Ref		ref	
**Other health conditions**^**§**^				
0-2	Ref		ref	
3 or more	1.75	1.12-2.73	1.72	1.01-2.93
**BMI Level**				
BMI 0 - < 25	Ref		ref	
BMI 25 - < 30	1.91	.93-3.93	1.91	.91-4.02
BMI >= 30	2.63	1.38-5.02	2.32	1.12-4.81
**Vigorous leisure activity**				
Unable to do or none	0.58	.34-.98	0.68	.40-1.13
Any per week	Ref		ref	
**Perceived health status**				
Fair/poor	0.56	.36-.86	0.52	.32-.84
Exc/VG/Good^||^	Ref		Ref	
**Year of survey**				
2002	NA		Ref	
2007	NA		0.66	.42-1.04

* CM use, the dependent variable for this study, was defined as use of any of the following in the past 12 months: acupuncture, Ayurveda, biofeedback, chelation therapy, chiropractic care, chiropractic or osteopathic manipulation, energy healing therapy/Reiki, folk medicine, hypnosis, massage therapy, movement based therapies, naturopathy, herbal supplements and other non-vitamin/non-mineral dietary supplements (NVNMDS), homeopathic treatment, diet-based therapies, traditional healers, yoga, tai chi, qi gong, and relaxation techniques.

† unadjusted or crude odds ratio.

‡ 95% confidence interval.

§ Adjusted Odds Ratio. The model controls for socio-demographic and health status variables significantly associated (p < 0.1) with the dependent variable in Table 2: race/ethnicity, region of residence, number of co-morbid conditions, BMI level, presence of coronary heart disease, the ability to perform vigorous leisure activity at least once per week and perceived health status, as well as for year of survey.

|| Excellent/Very Good/Good.

#### Types of CM therapies used

In exploratory analyses, we examined the prevalence of use of four categories of CM to treat type-2 diabetes, and their associations with diabetes severity. Of the four categories, diet-based interventions (35.19%, S.E. 5.11) were the most used group of therapies followed closely by non-vitamin/non-mineral dietary supplements (NVNMDS) (33.74%, S.E. 5.07), then more distantly by therapies categorized as “other” (21%, S.E> 4.55). Relatively few individuals used multiple CM therapies (9.86%, S.E. 3.02) to treat or manage their diabetes. Of the measures of diabetes severity, only use of diabetes prescription medications varied significantly by CM category (Table 5). Individuals who used only NVNMDS were more likely to use diabetes prescription medications than did individuals who used multiple CM therapies.

**Table 5:** Use of selected complementary medicine (CM) categories to treat/manage type-2 diabetes, by selected measures of diabetes severity

	CM Diets^*^n=44		**NVNMDS**^**†**^**n=55**		**Other CM**^**‡**^**n=29**		**Multi CM**^**§**^**n=13**		
	**Percent**^**||**^	**S.E.**^**¶**^	**Percent**^**||**^	**S.E.**^**¶**^	**Percent**^**||**^	**S.E.**^**¶**^	**Percent**^**||**^	**S.E.**^¶^	
**TOTAL**	35.19	5.11	33.74	5.07	21.21	4.55	9.86	3.02	
	Percent^#^	S.E. ^¶^	Percent^#^	S.E.	Percent^#^	S.E. ^¶^	Percent^#7^	S.E. ^¶^	P-value^††^
**Count of diabetes severity measures**^**‡‡**^									
0-2	81.97	6.85	66.86	6.7	56.69	11.7	88.89	10.4	0.195
>=3	18.09	6.85	33.14	6.7	43.31	11.7	11.11	10.4	
**DM duration(years**)									
1-< 5	40.5	6.96	30.76	7.5	34.57	13.8	63.31	15.3	0.109
5-<10	23.52	7.93	16.9	6.6	3.27	2.45	4.63	4.64	
>= 10	35.89	8.77	52.34	7.98	62.16	13.5	32.06	14.5	
**Diabetes medication use**									
None	36.25	10.4	7.99	3.57	5.75	5.6	64.71	13.6	0.009
Any	63.75	10.4	92.01	3.57	94.25	5.6	35.29	13.6	
**Functional limitation**									
No	84.88	5.21	78.88	5.24	60.37	11.6	81.73	12.3	0.25
Yes	15.12	5.21	21.12	5.24	39.63	11.6	18.27	12.3	
**Weak/failing kidneys**									
No	89.66	6.02	90.36	4.54	98.26	1.76	96.23	4.4	0.304
Yes	10.34	6.02	9.64	4.54	1.74	1.76	3.77	4.4	
**Coronary Heart Disease**									
No	94.77	3.09	91.08	4.39	89.7	6	100	0	0.139
Yes	5.23	3.09	8.92	4.39	10.3	6	0	0	
**Severe vision problems**									
No	74.67	8.21	82.59	4.75	69.29	10.0	85.7	9.88	0.603
Yes	25.33	8.21	17.41	4.75	30.71	10.0	14.3	9.88	
**Anxiety or Depression**									
No	75.38	7.78	79.01	5.66	57.35	13.3	73.45	12.1	0.609
Yes	24.62	7.78	20.99	5.66	42.65	13.3	26.55	12.1	

* Vegetarian diet, Macrobiotic diet, Atkins diet, Pritikin diet, Ornish diet, Zone diet, and South Beach.

† herbal supplements and other non-vitamin/non-mineral dietary supplements.

‡ acupuncture, Ayurveda, biofeedback, chelation therapy, chiropractic care, chiropractic or osteopathic manipulation, energy healing therapy/Reiki, folk medicine, hypnosis, massage therapy, movement based therapies, naturopathy, homeopathic treatment, traditional healers, yoga, tai chi, qi gong, and relaxation techniques

§ Any combination of multiple CM therapies.

|| The denominator used in the calculation of percentages was the number of adults with type-2 diabetes who used any CM therapy to treat/manage their diabetes. Estimates were age adjusted using the projected 2000 U.S. population as the standard population and using four age groups: 18–24 years, 25–44 years, 45–64 years, and 65 years and over.

¶ Standard Errors.

# The denominator used in the calculation of percentages was the number of adults with type-2 Diabetes who used the indicated type of CM therapy. Estimates were age adjusted using the projected 2000 U.S. population as the standard population and using four age groups: 18–24 years, 25–44 years, 45–64 years, and 65 years and over.

†† Chi-square analyses comparing use of the indicated CM therapies in individuals with Type-2 diabetes who use CM to treat/manage their diabetes

‡‡ Based on the following five measures: 5 or more years since time of diagnosis, use of insulin or oral hypoglycemic drugs, at least one functional limitation attributed to the diabetes, weak or failing kidneys, and severe vision problems.

A total of 12 different NVNMDS were used by individuals to treat or manage their diabetes: glucosamine, fiber or psyllium, fish oil or omega 3, flax seed oil, garlic supplements, ginseng, green tea pills, saw palmetto, cranberry pills, evening primrose, milk thistle, and lecithin. However, the numbers using each agent were too small to permit any demographic analyses.

Within the “Other” CM category, individuals used nine different therapies to treat or manage their diabetes: acupuncture, biofeedback, folk medicine, chiropractic care, homeopathy, naturopathy, relaxation techniques, tai chi, and yoga. Too few individuals used any specific “other” therapy to permit detailed analyses.

#### Reasons for CM use

Of those who used CM to treat or manage their diabetes, 20.13% (S.E. 3.87) said they did so because they believed conventional treatments did not help, while 21.3% (S.E. 4.17) did so because they felt conventional medical treatments were too expensive. No associations were seen between the response to these questions and measures of diabetes severity (data not shown).

#### Sensitivity analyses

Sensitivity analyses are presented in Table 6. While addition of anxiety/depression (as a diabetes complication) to the count measure of disease severity produced a small reduction in the strength of association between disease severity and use of CM (from AOR= 1.9 to AOR= 1.74), the association remained statistically significant. As an individual measure of diabetes severity, anxiety/depression was not associated with CM use. Stepping down the age cutoff for diagnosis of diabetes from <25 years of age to <10 years of age produced a slight reduction in the strength of the association between diabetes severity and use of CM (AOR=1.9 to AOR 1.79) that remained statistically significant.

**Table 6:** Sensitivity Analyses of associations between diabetes severity and use of complementary medicine (CM)* to treat/manage type-2 diabetes

	**UOR**^**†**^	**95% CI**^**‡**^	**AOR**^**§**^	**95% CI**^**‡**^
**Analysis includes all individuals coded as having type-2 diabetes**				
Count of diabetes severity measures includes anxiety or depression				
0-2 measures	Ref		Ref	
>=3 measures	1.16	0.77-1.77	1.64	1.07-2.52
Anxiety or Depression				
No	Ref		Ref	
Yes	1.05	.65-1.7	1.16	.64-2.12
**Alternate coding strategies for type-2 diabetes which vary the age at which diabetes was diagnosis**				
**<20 years old**				
Original count of diabetes severity measures^||^				
0-2 measures	ref		Ref	
>=3 measures	1.21	.80-1.83	1.78	1.13-2.81
**<15 years old**				
Original count of diabetes severity measures^||^				
0-2 measures	ref		Ref	
>=3 measures	1.18	.78-1.78	1.75	1.11-2.76
**<10 years old**				
Original count of diabetes severity measures^||^				
0-2 measures	ref		Ref	
>=3 measures	1.25	.84-1.85	1.79	1.14-2.80

* CM use, the dependent variable for this study, was defined as use of any of the following in the past 12 months: acupuncture, Ayurveda, biofeedback, chelation therapy, chiropractic care, chiropractic or osteopathic manipulation, energy healing therapy/Reiki, folk medicine, hypnosis, massage therapy, movement based therapies, naturopathy, herbal supplements and other non-vitamin/non-mineral dietary supplements (NVNMDS), homeopathic treatment, diet-based therapies, traditional healers, yoga, tai chi, qi gong, and relaxation techniques.

† unadjusted or crude odds ratio.

‡ 95% confidence interval.

§ Adjusted Odds Ratio. The model controls for socio-demographic and health status variables significantly associated (p < 0.1) with the dependent variables in Table 2: race/ethnicity, region of residence, number of co-morbid conditions, BMI level, the ability to perform vigorous leisure activity at least once per week and perceived health status, as well as for year of survey.

|| Based on the following six measures: 5 or more years since time of diagnosis, use of insulin or oral hypoglycemic drugs, at least one functional limitation attributed to the diabetes, weak or failing kidneys, Coronary Heart Disease, and severe vision problems.

## Discussion

To our knowledge, this is the first report to examine the association between the use of CM and severity of diabetes in a large, nationally representative population sample. We found that only a small proportion of individuals coded with type-2 diabetes used CM to treat or manage their diabetes (3.4%). However, in this small cohort, persons with the most severe disease (as assessed by having 3 or more measures of diabetes severity) had almost twice the odds of using a complementary therapy as those with less severe disease. This association was relatively insensitive to the inclusion of anxiety/depression as a diabetes complication, and to changes in the age at diagnosis cutoff for differentiating type-1 and type-2. Furthermore, we found that persons who had diabetes 10 or more years and those who had a functional limitation resulting from their diabetes were more likely to use at least one CM therapy. Our study could not determine whether the use of CM is a direct result of diabetes severity or whether other unmeasured and uncontrolled factors motivate use.

While our observation that 30.9% of individuals with type-2 diabetes used CM for any reason is consistent with early reports [11,12], our finding that only 3.4% of individuals with type-2 specifically use CM to treat or manage their diabetes is substantially lower than that reported by Yeh et al., 2002 (20%) [11]. Although it is possible some of these discrepancies reflect real changes in use over time, it is more likely the discrepancies reflect methodological differences in the two surveys. In calculating their prevalence of CM use in 1997, Yeh and colleagues [11] included four types of therapies not included in the present analyses: folk remedies (2.9%)(included in the 2002 but not 2007 NHIS), commercial diets (6.4%), high dose vitamins (1.7%) and self-help groups (2.3%). In addition, Yeh et al. [11] found higher use of herbal medicines to treat or manage diabetes then we did for NVNMDS in the present study, 6.7% vs. 1.1%, respectively. This discrepancy is most likely explained by that fact that Yeh et al. [11] employed a global question on use of “herbal medicine”, while in the NHIS participants were asked if they used specific NVNMDS on a list.

While the inverse association between the use of CM and the use of prescription diabetes medications failed to reach significance in either the unadjusted or adjusted logistic regression models, some comment is still warranted given concerns of herb-drug interactions [38] or the substitution of CM therapies for proven conventional therapies for diabetes [39]. There are two competing interpretations of our observation: 1) those using CM are more likely to forego needed conventional treatment; or 2) individuals with less severe disease not requiring medical interventions are more likely to use CM. When examining CM categories, we observed that diabetes medication use predominates in CM users across categories except in participants using multiple CM therapies (only 35.3% of whom used diabetic medications). Except for multiple CM users, the present CM category data are consistent with a regional survey of older rural adults diagnosed with diabetes (N= 679) [40], where no difference in diabetes medication adherence was found between those who used CM and those who didn’t.

Consistent with our observation that most individuals (77%) who use CM for their diabetes also use conventional diabetes prescription medications are the data of Garrow and Egede [41] who found that individuals with diabetes using CM (for any reason) were more likely to receive regular preventive care than were individuals with diabetes who did not use CM. These quantitative findings are supported by qualitative research [8] suggesting that individuals with diabetes who try CM were highly motivated to control their diabetes and to try all methods available to them, and did not use CM as a substitute for conventional care.

We are aware of only one other article that discussed demographic characteristics of individuals in the United States using CM specifically to treat or manage their diabetes [7]. In this survey of rural older adults in North Carolina, it was found that in those using CM for their diabetes, home remedies were the prevalent type of CM therapy used, with their use associated with race/ethnicity and the number of co-morbidities. In the present study, we also found that the number of co-morbidities was associated with the overall use of CM but did not see a relationship between CM use and race/ethnicity. Unfortunately, the NHIS did not specifically look at use of home remedies, which may explain the differences in our findings from Arcury et al. [7].

Our analyses revealed an interesting paradox in that persons with more severe diabetes are more likely to use CM to treat/manage their diabetes, but persons with poorer perceived general health status were less likely to use CM (OR 0.56, 95% CI .33-.86). Using data from the 2007 NHIS, Nguyen and colleagues [42] observed a similar paradox in the general population where individuals “using CAM were more likely to have chronic illness . . . yet also were more likely to report that their health status was excellent and better than the prior year.” Nguyen et al., [42] suggested two possible reasons for the paradox, both of which also apply to our analyses: 1) different timeframes for the health status questions (current status) and illness self-reports (previous 12-months); 2) positive expectations of CM use may positively impact perceived health status.

We found that relatively few individuals with diabetes who used CM for their diabetes used more than one type of CM therapy (<10%). This is in contrast to the general population in 2007 (unpublished observation) where 41.4% of CM users used more than one type of therapy, or in 2002 or 1997 where, respectively, 33.4% [43] and 54% [44] of CM users used more than one type of therapy (as in the present analysis, these studies counted use of multiple NVNMDS or multiple diet-based therapies only once each).

This study has several limitations. First, the variables being investigated were self-reported. The scientific literature suggests that most people tend to under-report negative health behaviors [45]. Hence, the effects of alcohol consumption [46,47] or smoking behavior [48] may have been underreported in this study. Second, the cross-sectional nature of the data prevents us from establishing whether the diagnosis of diabetes actually occurred prior to and resulted in the diseases/conditions we considered as complications of diabetes. Thus, it is likely that for some unknown percent of participants, non-complications were misclassified as complications. From our data, we cannot predict how such misclassification would impact on our results. These cross-sectional data also do not let us investigate the possibility of cohort effects and secular trends in the associations between diabetes severity and CM use. Third, the lack of confirmed diagnoses of type-1 and type-2 diabetes makes some degree of misclassification likely. Based on our criteria for differentiating between type-1 and type-2 diabetes and on published NHANES data [49], we can predict that 7-9% of the cases we classified as type-2 were in fact type-1. Conversely, given the increasing prevalence of type-2 diabetes in youth, we can predict that some unknown proportion of individuals we classified as probably having type-1 diabetes actually had type-2. We sought to more completely capture the population of individuals with type-2 diabetes by lowering the cutoff age for differentiating type-1 and type-2. We found that lowering the cutoff age to less than 10-years old, a cutoff likely to capture almost all, if not all individuals with type-2 diabetes [34], did not substantially change our findings. However, clearly our study needs to be replicated in a dataset that incorporates confirmed physician diagnosis of diabetes by type and also contains information on the use of CM. Fourth, it is possible that additional measures of diabetes severity (as well as other unexplored factors) explain more of the observed relationships. However, the six measures employed are predictors of mortality and morbidity in diabetes [50,51]. Fifth, because our primary focus was to identify factors associated with the use, versus nonuse, of CM, a dichotomous dependent variable was utilized for the primary analysis. By doing so, information on frequency and/or intensity of use was lost. It may be that substantial differences exist between heavy and light users of one or more CM modalities [44]. Sixth, it has been found that the use of specific types of CM therapies is associated with specific personality styles [52]. These associations might confound our results if specific personality styles (e.g., “openness” or “control”) are also related to better diabetes control. Finally, despite combining data from two very large national surveys in the United States, the number of respondents using CM specifically to treat their diabetes was small (141 respondents, equivalent to 3.4% of individuals with type-2 diabetes). While this may have increased the chance of type-2 error (false negatives) in our analyses and certainly prevented assessment of individual CM therapies, it does not change our confidence in the positive associations we did observe.

## Conclusions

Our results demonstrate that individuals with more severe diabetes are more likely to use CM independent of sociodemographic factors. Further studies are essential to determine if CM therapies, either alone or in combination with conventional approaches, actually improve clinical outcomes when used to treat/manage diabetes. Also warranted are studies investigating the cause and effect relationships underlying the associations between diabetes severity and CM use. Knowledge of these specific patterns of use may contribute to tailoring health education programs for diabetes. For health care providers, understanding the motivations behind a patient’s use of CM may assist in the design of an optimal treatment plan [53]. The fact that users of CM appear willing to take active control of their health [54-56], suggests that this group of individuals with diabetes may well be open to additional recommendations toward managing their disease.

## Competing interests

All authors declare they have no competing interests.

## Authors’ contributions

RLN and BJS contributed to the study conception and design. DBC programmed the study. RLN and DBC analyzed the data. RLN and NK interpreted the results. RLN developed the first draft of the manuscript. DBC, BJS, and NK reviewed and edited the manuscript. The final draft for submission was read and approved by all authors. The findings and conclusions in this report are those of the authors and do not necessarily represent the official position of the National Institutes of Health. All authors have read and approved the final manuscript.

## Pre-publication history

The pre-publication history for this paper can be accessed here:

http://www.biomedcentral.com/1472-6882/12/193/prepub
